# Supplementary material for: Identifying therapeutic targets by combining transcriptional data with ordinal clinical measurements
Source: Nat Commun. 2017 Sep 20;8:623. doi: 10.1038/s41467-017-00353-6 (PMC5606996; doi:10.1038/s41467-017-00353-6)
Supplement: Supplementary file 1 — Supplementary Information [file 41467_2017_353_MOESM1_ESM.pdf]

File name: Supplementary Information
Description: Supplementary figures and supplementary tables.

File name: Supplementary Data 1
Description: The list of phenotype–associated genes in HD.

File name: Peer review file
Description:

Down-regulated genes		
**Description**	P-value	FDR q-value
**synaptic transmission**	1.15E-09	4.32E-06
**cell communication**	4.55E-09	8.52E-06
**single organism signaling**	3.90E-08	4.87E-05
**signaling**	3.90E-08	3.65E-05
**cell-cell signaling**	7.12E-08	5.34E-05
**cellular ion homeostasis**	5.51E-07	3.44E-04
**cation transport**	1.52E-06	8.15E-04
**ion transport**	2.57E-06	1.20E-03
**ion homeostasis**	3.99E-06	1.66E-03
**cellular homeostasis**	3.99E-06	1.49E-03
**cellular chemical homeostasis**	5.49E-06	1.87E-03
**Up-regulated genes**		
**Description**	P-value	FDR q-value
**nucleic acid metabolic process**	2.37E-14	8.88E-11
**RNA metabolic process**	6.97E-14	1.31E-10
**nitrogen compound metabolic process**	3.21E-12	4.01E-09
**organic cyclic compound metabolic process**	3.21E-12	3.01E-09
**RNA biosynthetic process**	7.12E-12	5.34E-09
**heterocycle metabolic process**	1.61E-11	1.00E-08
**cellular nitrogen compound metabolic process**	1.82E-11	9.74E-09
**transcription, DNA-dependent**	1.14E-09	3.88E-07
**primary metabolic process**	1.25E-07	2.34E-05
**regulation of gene expression**	1.31E-07	2.34E-05
**regulation of RNA metabolic process**	2.50E-07	3.90E-05

Supplementary Table 1.
The GO enrichment of inferred phenotype-associated genes in HD.

ID	Type	Sex	CAG repeat number	Age at onset	Age at death	Postmortem interval
HD_01	HD	Male	45	42	53	14.55
HD_02	HD	Male	46	40	54	20.08
HD_03	HD	Male	41	45	74	9.11
HD_04	HD	Male	44	38	51	19.67
HD_05	HD	Male	50	25	40	20.50
HD_06	HD	Male	46	39	51	18.88
HD_07	HD	Male	44	50	65	24.58
HD_08	HD	Male	42	36	53	17.00
HD_09	HD	Male	50	28	42	21.25
HD_10	HD	Male	47	25	49	18.62
HD_11	HD	Male	44	43	57	21.50
HD_12	HD	Male	47	40	62	22.50
C_01	Control	Male			68	25.00
C_02	Control	Male			56	17.32
C_03	Control	Male			73	18.02
C_04	Control	Male			66	24.55
C_05	Control	Male			55	24.83
C_06	Control	Male			67	23.12
C_07	Control	Male			70	25.83
C_08	Control	Male			56	18.51

Supplementary Table 2.
Information about samples from human postmortem cortical brain tissue.

Human	STHDH cell line	12 w R6/2	CHL2	R6/1
B4GALT6, Beta-1,4-Galactosyltransferase 6	***Smpd3***, Sphingomyelin Phosphodiesterase 3	***Smpd3***, Sphingomyelin Phosphodiesterase 3	***Sgpp2***, Sphingosine-1-Phosphate Phosphatase 2	***Ppap2b***, Phosphatidic Acid Phosphatase Type 2A
SGPL1, Sphingosine-1-Phosphate Lyase 1	***Gla***, Galactosidase Alpha	***Neu2***, Neuraminidase 2	***Smpd3***, Sphingomyelin Phosphodiesterase 3	***Ugt8a***, UDP Glycosyltransferase 8
SMPD3, Sphingomyelin Phosphodiesterase 3	***Sgpl1***, Sphingosine-1-Phosphate Lyase 1	***Ppap2b***, Phosphatidic Acid Phosphatase Type 2A	***Neu2***, Neuraminidase 2	***Neu2***, Neuraminidase 2
SGPP2, Sphingosine-1-Phosphate Phosphatase 2	***Arsa***, Arylsulfatase A	***Sgpp2***, Sphingosine-1-Phosphate Phosphatase 2		***Smpd3***, Sphingomyelin Phosphodiesterase 3
KDSR, 3-Ketodihydrosphingosine Reductase	***Ppap2a***, Phosphatidic Acid Phosphatase Type 2A			***B4galt6***, Beta-1,4-Galactosyltransferase 6
PPAP2A, Phosphatidic Acid Phosphatase Type 2A	***Sgpp1***, Sphingosine-1-Phosphate Phosphatase 1			
PPAP2B, Phosphatidic Acid Phosphatase Type 2B	***Sptlc2***, Serine Palmitoyltransferase Long Chain Base Subunit 2			
SPHK1, Sphingosine Kinase 1	***Sphk1***, Sphingosine Kinase 1			

Supplementary Table 3.
The list of differentially expressed genes involved in sphingolipid metabolism and the corresponding HD models.

A cluster		
**Description**	P-value	FDR q-value
**response to interferon-beta**	1.05E-18	1.41E-14
**response to stress**	1.14E-18	7.72E-15
**regulation of immune system process**	2.25E-12	3.79E-09
**innate immune response**	4.26E-12	6.39E-09
**response to cytokine**	9.56E-11	6.14E-08
**response to chemical**	1.67E-10	1.03E-07
**B & C cluster**		
**Description**	P-value	FDR q-value
**cell projection organization**	1.42E-11	2.02E-07
**regulation of nervous system development**	3.28E-07	2.90E-04
**regulation of neuron projection development**	3.34E-07	2.78E-04
**regulation of neurogenesis**	4.39E-07	2.96E-04
**biological adhesion**	2.13E-06	1.04E-03
**regulation of axonogenesis**	1.52E-05	5.39E-03
**actin filament-based process**	4.70E-05	1.28E-02
**regulation of axon extension**	7.76E-05	1.80E-02
**regulation of protein kinase activity**	8.54E-05	1.92E-02
**transmembrane receptor protein tyrosine kinase signaling pathway**	8.57E-05	1.90E-02
**regulation of small GTPase mediated signal transduction**	1.03E-04	2.15E-02
**regulation of Ras protein signal transduction**	5.64E-04	7.41E-02
**regulation of Rho protein signal transduction**	6.08E-04	7.77E-02

Supplementary Table 4.
Gene Ontology enrichments of the genes from cluster A, B and C.

Component of 146 genes with IEZ > 0

Description	P-value	FDR q-value
cytoskeleton	1.21E-06	3.91E-04
cell projection part	5.75E-05	1.54E-02
membrane-bounded organelle	1.40E-04	2.81E-02
neuron part	2.24E-04	3.60E-02
cell projection	3.06E-04	3.52E-02
neuronal cell body	5.74E-04	5.14E-02

Biological function of 146 genes with IEZ > 0

Description	P-value	FDR q-value
protein binding	3.08E-09	1.47E-05
ion binding	4.80E-08	1.15E-04
anion binding	1.89E-07	3.02E-04
Ras guanyl-nucleotide exchange factor activity	1.17E-06	1.12E-03
nucleoside phosphate binding	2.96E-06	2.36E-03
cytoskeletal protein binding	5.92E-06	3.54E-03

Supplementary Table 5.

Functional and component enrichment of genes that increase in expression after treatment of STHdh Q111 cells with DOP compared to untreated cells, and that also show an increase in H3K9ac in their promoters.

Ordinal Categories	Vonsattel grade	No. of samples
**1**	**Control**	**32**
**2**	**HD Grade 0 or 1**	**16**
**3**	**HD Grade 2**	**16**
**4**	**HD Grade 3 or 4**	**6**

Supplementary Table 6.
Four ordinal categories for control and HD samples, representing the degree of neurodegeneration. Additionally, the table shows the number and Vonsattel grade of samples in each category.

Supplementary Figures:

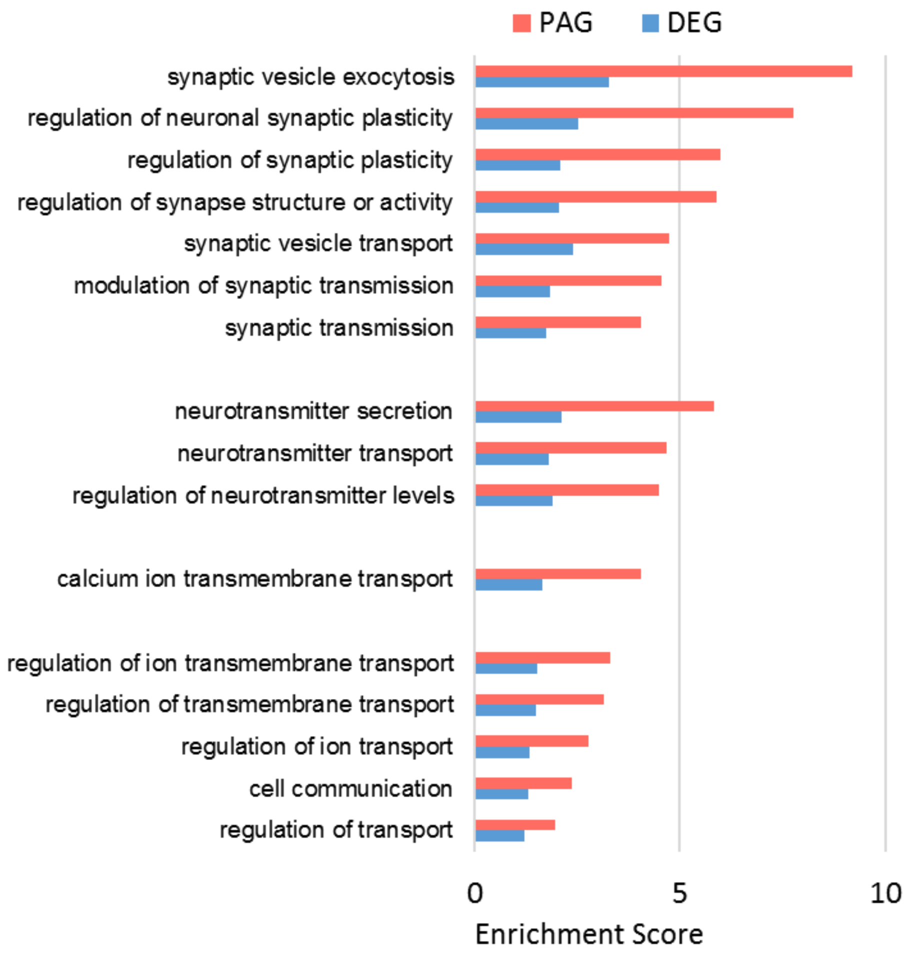

Supplementary Figure 1.
Comparison of the enrichment of phenotype-associated genes (PAGs) and differentially expressed genes (DEGs) in the known dysregulated biological processes in HD. The horizontal bars show the enrichment value of biological processes.

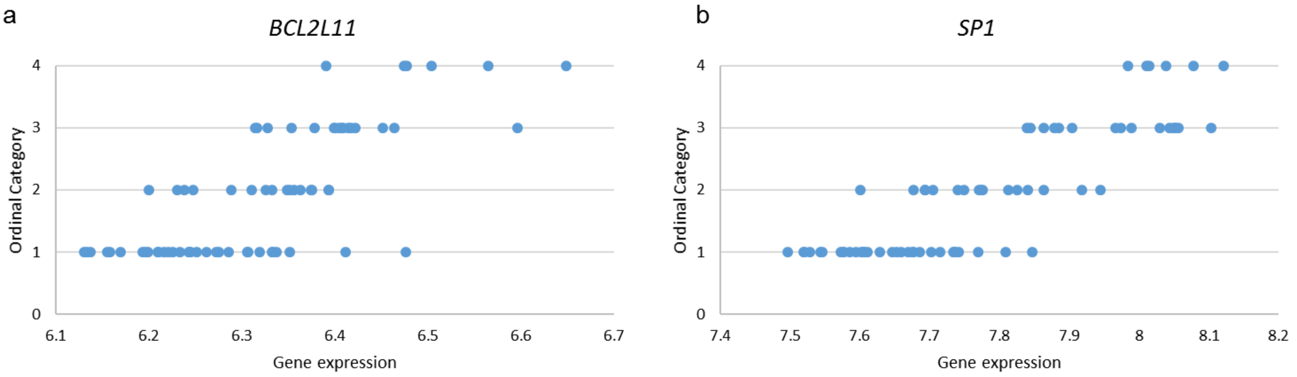

Supplementary Figure 2.
The expression of *BCL2L11* (a) and *SP1* (b), which are the top two ranked phenotype associated genes, with the ordinal category of neurodegeneration. Each dot shows the expression of the gene from a sample with ordinal neurodegenerative grade y.

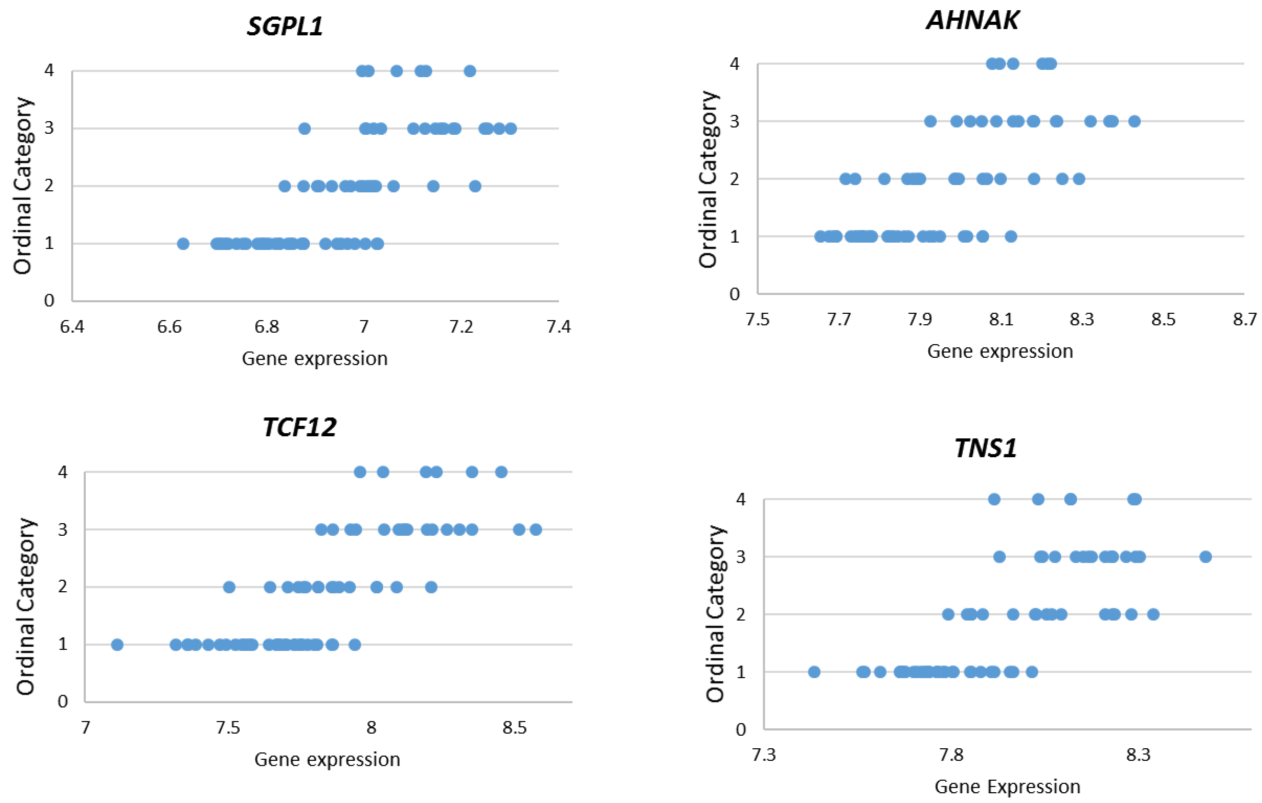

Supplementary Figure 3.

The expression of high-ranked up-regulated genes, including *SGPL1*, *AHNAK*, *TCF12*, and *TNS1*, that are also differentially expressed in STHdh cell line model, with the ordinal category of neurodegeneration. Dots display the expression of genes from samples with ordinal neurodegenerative grade y.

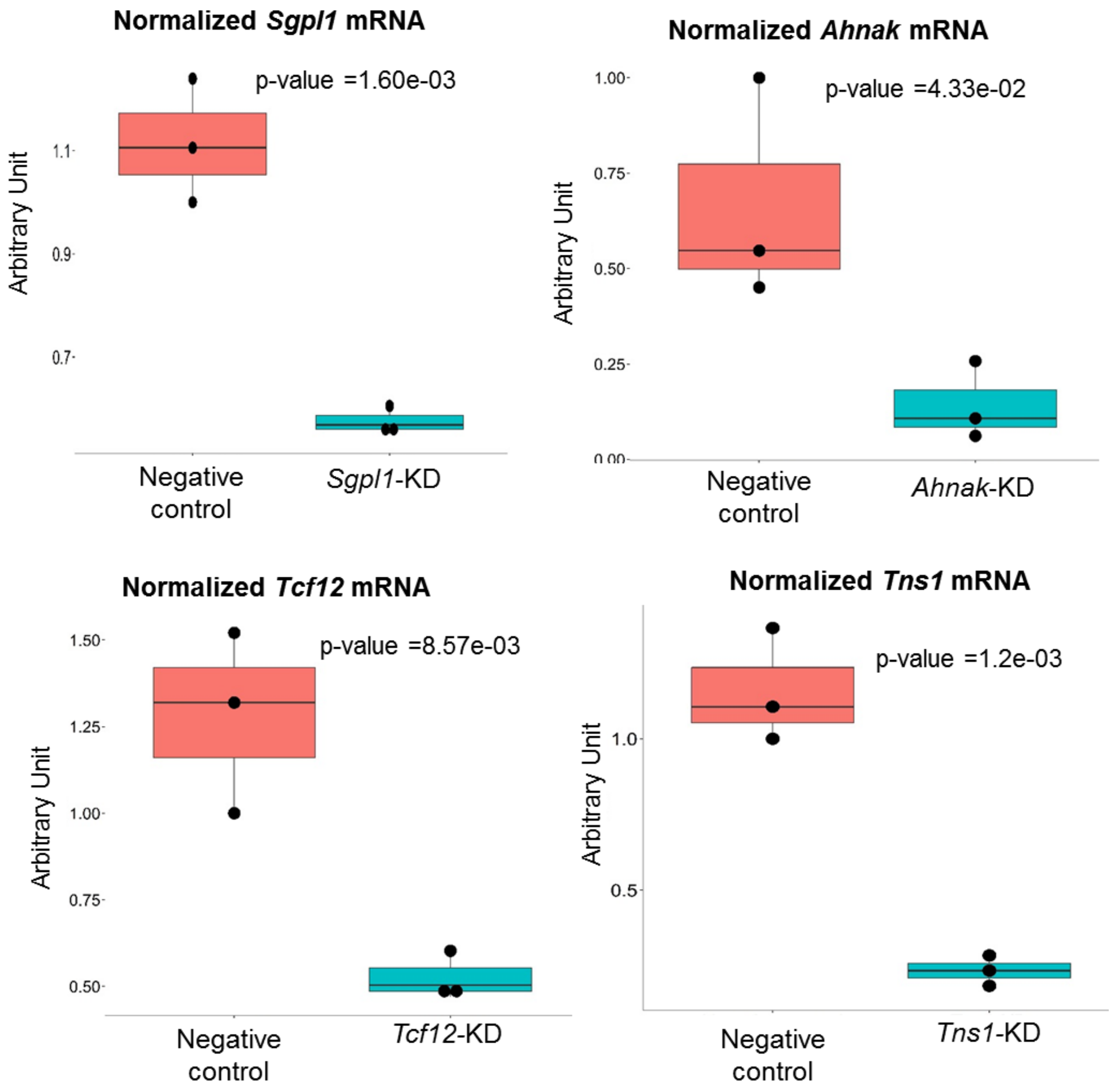

Supplementary Figure 4.
The plots show a significant decrease in the normalized mRNA levels of *Sgpl1* (p-value=1.6×10^{-3}), *Ahnak* (p-value=4.3×10^{-2}), *Tcf12* (p-value=8.57×10^{-3}), and *Tns1* (p-value=1.2×10^{-3}) after knock-down experiments. The boxes show the distribution of relative mRNA levels, measured by real time RT-PCR and expressed as the $2^{-\Delta\Delta CT}$ values. Two-tailed t-test were performed to calculate statistical significance. The first and the third quartiles of the data are displayed by the top and the bottoms of respective boxes and the median (second quartile value) is shown as a horizontal line within the box.

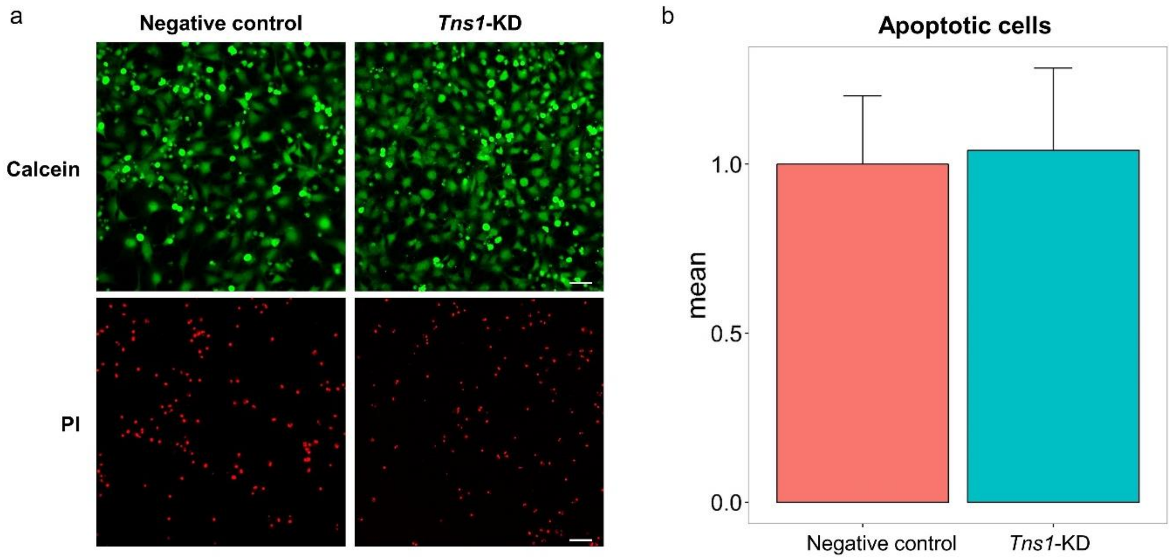

Supplementary Figure 5.
(a) Cell viability after knocking-down *Tns1* in STHdh Q111 cells compared to controls. Calcein (green) stains viable cells, and propidium iodide (PI – red) shows late apoptotic cells. Scale bar = 10 µm. (b) The number of apoptotic cells are represented as fold changes normalized to the control. Two-tailed t-test were performed to calculate statistical significance. The bar shows the mean value and the error bars indicate the standard deviation.

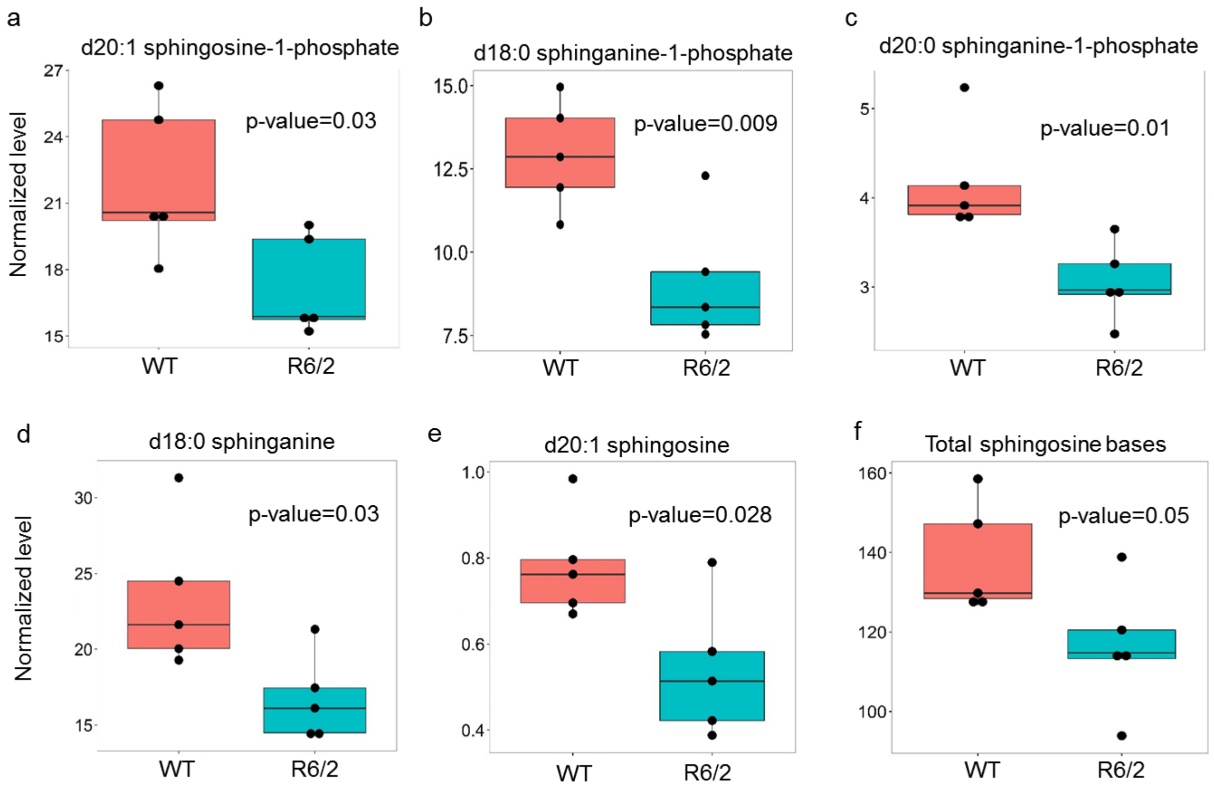

Supplementary Figure 6.

R6/2 model of HD exhibit decreased levels of sphingosine metabolites in striatum. The levels of several sphingolipids such as d20:1 sphingosine-1-phosphate (a), d18:0 sphinganine-1-phosphate (b), d20:0 sphinganine-1-phosphate (c), d18:0 sphinganine (d) d20:1 sphingosine (e) are significantly decreased in the striatum of R6/2 mice compared to wild-type controls. Additionally, total levels of sphingosine bases were decreased (f). All values are normalized to total protein input and are expressed as pmol/mg protein. Statistical significance was calculated using two-tailed t-test (n=5 per group). The boxplots display the distribution of the level of these sphingolipids. The first and the third quartiles of the data are displayed by the top and the bottoms of respective boxes and the median (second quartile value) is represented as a horizontal line.

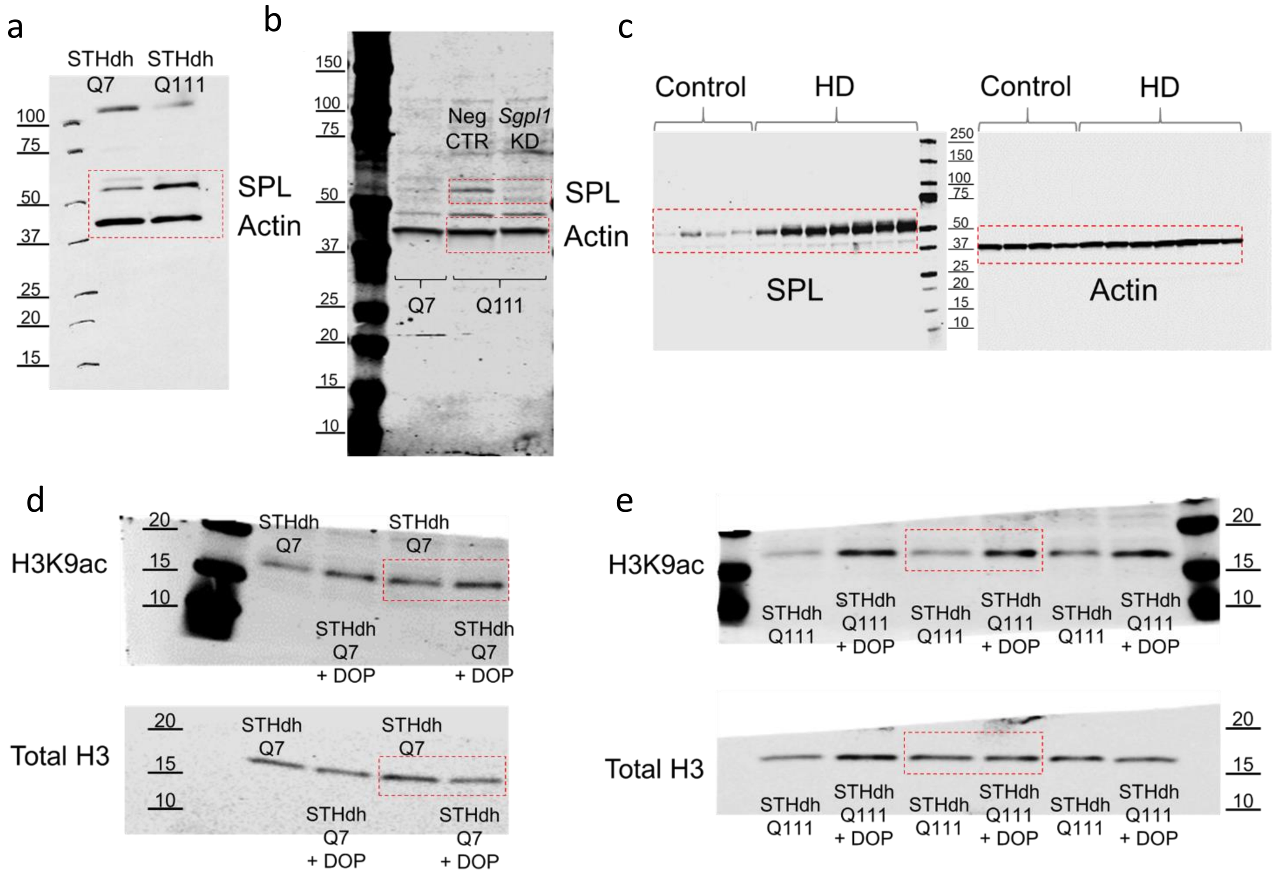

Supplementary Figure 7.

This image shows the uncropped version of Fig. 4a (a), Fig. 4b (b), Fig 4c (c), Fig. 8a for STHdh Q7 (d) and STHdh Q111 (e). The red boxes indicate the cropped area used to generate the figures in the main article.
